# Association of Family Ratings of Quality of End-of-Life Care With Stopping Dialysis Treatment and Receipt of Hospice Services

**DOI:** 10.1001/jamanetworkopen.2019.13115

**Published:** 2019-10-11

**Authors:** Claire A. Richards, Paul L. Hebert, Chuan-Fen Liu, Mary Ersek, Melissa W. Wachterman, Leslie L. Taylor, Lynn F. Reinke, Ann M. O’Hare

**Affiliations:** 1 Health Services Research and Development, Seattle-Denver Center of Innovation for Veteran-Centered and Value-Driven Care, US Department of Veterans Affairs, Seattle, Washington; 2 Department of Health Services, School of Public Health, University of Washington, Seattle; 3 Corporal Michael J. Crescenz VA Medical Center–Philadelphia, Philadelphia, Pennsylvania; 4 School of Nursing, Perelman School of Medicine, University of Pennsylvania, Philadelphia; 5 Veterans Affairs Boston Healthcare System, Boston, Massachusetts; 6 Dana-Farber Cancer Institute, Brigham and Women’s Hospital, Harvard Medical School, Boston, Massachusetts; 7 School of Nursing, Department of Biobehavioral Nursing and Health Informatics, University of Washington, Seattle; 8 University of Washington School of Medicine, Seattle

## Abstract

**Importance:**

Approximately 1 in 4 patients receiving maintenance dialysis for end-stage renal disease eventually stop treatment before death. Little is known about the association of stopping dialysis and quality of end-of-life care.

**Objectives:**

To evaluate the association of stopping dialysis before death with family-rated quality of end-of-life care and whether this association differed according to receipt of hospice services at the time of death.

**Design, Setting, and Participants:**

This survey study included data from 3369 patients who were treated with maintenance dialysis at 111 Department of Veterans Affairs medical centers and died between October 1, 2009, to September 30, 2015. Data set construction and analyses were conducted from September 2017 to July 2019.

**Exposure:**

Cessation of dialysis treatment before death.

**Main Outcomes and Measures:**

Bereaved Family Survey ratings.

**Results:**

Among 3369 patients included, the mean (SD) age at death was 70.6 (10.2) years, and 3320 (98.5%) were male. Overall, 937 patients (27.8%) stopped dialysis before death and 2432 patients (72.2%) continued dialysis treatment until death. Patients who stopped dialysis were more likely to have been receiving hospice services at the time of death than patients who continued dialysis (544 patients [58.1%] vs 430 patients [17.7%]). Overall, 1701 patients (50.5%) had a family member who responded to the Bereaved Family Survey. In adjusted analyses, families were more likely to rate overall quality of end-of-life care as excellent if the patient had stopped dialysis (54.9% vs 45.9%; risk difference, 9.0% [95% CI, 3.3%-14.8%]; *P* = .002) or continued to receive dialysis but also received hospice services (60.5% vs 40.0%; risk difference, 20.5% [95% CI, 12.2%-28.9%]; *P* < .001).

**Conclusions and Relevance:**

This survey study found that families rated overall quality of end-of-life care higher for patients who stopped dialysis before death or continued dialysis but received concurrent hospice services. More work to prepare patients for end-of-life decision-making and to expand access to hospice services may help to improve the quality of end-of-life care for patients with end-stage renal disease.

## Key Points

**Question:**

What is the association of family-rated quality of end-of life care with stopping maintenance dialysis treatments and receipt of hospice services before death?

**Findings:**

In this survey study of family members of patients with end-stage renal disease who were undergoing maintenance dialysis, the adjusted predicted probability of family rating the quality of end-of-life care as excellent was higher for patients who stopped dialysis before death than for patients who did not stop dialysis (54.9% vs 45.9%). Among patients who did not stop dialysis, receipt of hospice services was associated with a higher probability of the patient’s family rating the quality of end-of-life care as excellent (60.5% vs 40.0%).

**Meaning:**

Preparing patients for end-of-life decision-making and improving access to concurrent receipt of dialysis and hospice services may help to improve the quality of end-of-life care for patients undergoing dialysis for end-stage renal disease.

## Teaser

This survey study examines the association of family-rated quality of end-of-life care with stopping dialysis treatment and receipt of hospice services among veterans with end-stage renal disease.

## Introduction

Many of the technological advances in medicine during the last half century have profoundly altered the nature of the dying process and the timing of death.^1^ In many instances, treatments intended to prolong life have the “double effect of prolonging life and prolonging dying.”^1^ The more widespread availability of technologies, such as dialysis, mechanical ventilation, artificial nutrition, and other life-sustaining interventions, can delay death but also raise ethically challenging questions about the value of life and whether and when to withhold or stop these treatments.^2,3,4^

To our knowledge, there is little currently known about the association of stopping life-sustaining treatments with the quality of end-of-life care. Prior studies have described the experiences of family members during or after their involvement in decisions to stop short-term^5,6,7,8,9,10,11^ and long-term^12,13,14,15^ life support. However, to our knowledge, only a small number of studies have evaluated the quality of end-of-life care as a function of whether life support was stopped prior to death.^12,16^ A study by Gries et al^16^ reported greater satisfaction with decision-making support among family members of patients in whom life support had been stopped in the intensive care unit (ICU) compared with those in whom life support was continued. However, a study by Cohen et al^12^ found that family members of patients who stopped maintenance dialysis were no less likely than those of patients who continued treatment to report that the patient had pain in the last week of life, had a peaceful death, or died with dignity but were more likely to report that the death was anticipated and that the patient’s wishes were followed.

In the United States, approximately 500 000 patients are currently receiving maintenance dialysis for treatment of end-stage renal disease (ESRD).^17,18^ Approximately 1 in 4 of these patients (or their families and clinicians) will ultimately decide to stop dialysis before death.^17,19^ In general, the decision to stop dialysis is often coupled with the decision to enroll in hospice, with much lower rates of hospice use noted among patients who continue dialysis treatments than among those who stop treatment before death.^17,20^ While treatment with maintenance dialysis among patients with advanced kidney disease has been associated with less favorable and receipt of hospice services has been associated with more favorable family ratings of end-of-life care,^21^ to our knowledge, little is known about the association of stopping dialysis with perceived quality of end-of-life care and the extent to which this might vary according to whether patients received hospice services.

Among a national cohort, we compared the quality of end-of-life care as rated by surviving family members of veterans receiving maintenance dialysis according to whether they had stopped dialysis treatments before death. We conducted a post hoc analysis to evaluate whether this association differed by receipt of hospice services at the time of death.

## Methods

This report was conducted according to the Strengthening the Reporting of Observational Studies in Epidemiology (STROBE) reporting guideline and the American Association for Public Opinion Research (AAPOR) reporting guideline. The US Department of Veterans Affairs (VA) Central Institutional Review Board approved this study and granted a waiver of informed consent because the patients were deceased. Data analyses were conducted from September 2017 to July 2019.

### Data Sources and Study Population

As previously described,^21^ we used VA administrative and clinical data to assemble a national cohort of veterans with advanced kidney disease between January 1, 2000, and December 31, 2014. We obtained linked data for cohort members from the US Renal Data System, a national registry for ESRD; VA inpatient, outpatient, and Fee Basis files; and Medicare Institutional and Carrier files.^22^ We obtained information on family-rated quality of end-of-life care from the Bereaved Family Survey (BFS). As part of a systemwide quality improvement initiative, this survey is administered between 6 and 10 weeks after death by the VA’s Veteran Experience Center to the next of kin of all eligible patients who die in VA inpatient facilities, including intensive care, acute care, dedicated palliative and hospice care units, and VA nursing homes.

**Figure 1.**
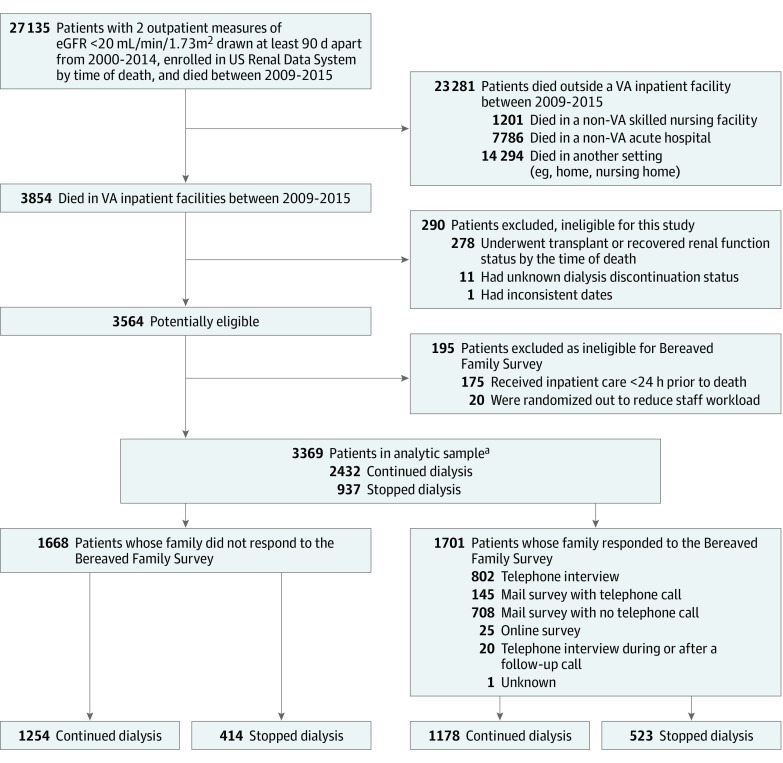
Flowchart for Cohort Selection eGFR indicates estimated glomerular filtration rate; VA, US Department of Veterans Affairs. ^a^Analytic sample includes both respondents and nonrespondents; models were adjusted for nonresponse using demographic and clinical characteristics and patterns of care of nonrespondents.

We conducted a retrospective observational study^21^ among those patients who died in a VA inpatient facility between October 1, 2009, and September 30, 2015, and appeared in the US Renal Data System registry as having received maintenance dialysis. Among these, we excluded patients who had recovered kidney function or had a functioning kidney transplant at the time of death and those who were missing information on whether they had stopped maintenance dialysis before death (Figure 1). We also excluded patients whose family members were not eligible to complete the BFS.^21^

### Exposure Variables

Patients were classified as having stopped dialysis before death based on information from the US Centers for Medicare & Medicaid Services (CMS)-2746 End-Stage Renal Disease Death Notification Form. The form is completed by a nephrologist after the patient’s death and includes questions about whether renal replacement therapy was discontinued prior to death and the circumstances under which dialysis was discontinued, including transplant failure, graft failure, acute medical complications, chronic failure to thrive, and other reasons. An affirmative response to the question about discontinuation does not necessarily indicate that stopping dialysis was the cause of death or that the patient died of uremia, only that there was a decision to stop dialysis before the patient died.^19^

To assess the validity of the CMS-2746 item on dialysis discontinuation among members of our cohort, we reviewed electronic health records for a random sample of 225 cohort members using Lucene text-search software (Apache Software Foundation) to capture documentation related to decisions to stop dialysis in text integration utilities notes from the VA’s Corporate Data Warehouse. Consistent with prior work to test the validity of the CMS-2746 item on treatment discontinuation,^23^ the criterion standard for comparison was the presence of documentation in patients’ electronic health records indicating that a decision had been made to stop dialysis at least 24 hours before death.

### Family Perceptions of End-of-Life Care

The BFS is a validated National Quality Forum–endorsed measure designed to ascertain family members’ perceptions of the quality of end-of-life care during the patient’s final month of life.^21,24,25,26,27^ We included the BFS Performance Measure, which is a single item that asks respondents to rate the patient’s overall quality of care as excellent, very good, good, fair, or poor. We also examined 10 individual items pertaining to communication, emotional and spiritual support, and pain management.^28^ To facilitate comparisons with earlier studies using the BFS, responses for all items other than pain were dichotomized as the best possible vs all other responses.^29,30,31,32^ How often the patient was uncomfortable from pain was dichotomized as never or sometimes vs usually or always.

### Covariates

#### Patient and Facility Characteristics

We ascertained each cohort member’s age at the time of death, sex, and fiscal year of death from the VA Vital Status files. Race was preferentially ascertained from the VA Corporate Data Warehouse; these data were acquired from self-report or proxy or entered by a VA enrollment coordinator or clerk.^33^ Missing data fields were populated first with race data from Medicare beneficiary files and then US Renal Data System registry data. Race was included as a covariate because it has been found to be associated with stopping dialysis and with satisfaction with end-of-life care.^17,29^ Information on the relationship of the next of kin invited to participate in the survey and the complexity of the facility in which the patient died (based on factors including patient volume and risk, presence of teaching and research, and available clinical services) was obtained from the Veteran Experience Center. Patients were assigned to a US Census division based on the state location of the facility in which they died.^34^ We ascertained all comorbidities that are included in the Charlson Comorbidity Index (revised by Quan et al^35^ for administrative data and modified as described previously^17,21,36^) using VA administrative data and Medicare claims during the year before death.

#### Hospice Services

We determined whether patients were receiving VA inpatient hospice services based on their assigned treating specialty at the time of death.^21^ Hospice care in VA inpatient facilities is primarily delivered in dedicated palliative care and hospice units but can be delivered in other settings, such as nursing homes, acute care wards, or ICUs.

#### End-of-Life Treatment Intensity

As described previously,^21,24,37,38^ we included the following 4 measures of high-intensity treatment, which have been found to be associated with lower quality of life or satisfaction with end-of-life care: (1) 2 or more weeks spent in the hospital within the last 90 days of life, (2) ICU admission within the last 30 days of life, (3) receipt of at least 1 intensive procedure within the last 30 days of life (ie, intubation and mechanical ventilation, cardiopulmonary resuscitation, feeding tube placement, enteral nutrition, tracheostomy),^22^ and (4) death in the ICU.

### Statistical Analysis

We used analysis of variance, *t* tests, Pearson χ^2^ tests, and Fisher exact tests to compare the characteristics of patients who did or did not stop dialysis before death and whose family members did or did not respond to the survey. We fit unadjusted and adjusted logistic regression models to examine the association of stopping dialysis with family-rated quality of end-of-life care. These analyses were adjusted for patients’ age (as a continuous variable), race, sex, the relationship of the next of kin to the patient, comorbid conditions, region, facility complexity, fiscal year of death, measures of high-intensity treatment, and receipt of hospice services. To evaluate whether hospice services might modify the association of stopping dialysis with quality of end-of-life care, an interaction term for hospice and stopping dialysis was included in all adjusted models. For all analyses, we used robust estimates of variance to adjust for facility-level clustering and calculated the predicted probabilities and average marginal effects for factors of interest using the distribution of covariates in the sample.

We accounted for survey unit and item nonresponse bias (eTable 1 and eTable 2 in the Supplement) in all adjusted models using nonresponse propensity weighting and multiple imputation by chained equations, respectively (eAppendix in the Supplement).^21,39,40,41,42^ We used SAS statistical software version 9.4 (SAS Institute) to construct the data set, Stata statistical software version 15 (StataCorp) and R version 3.5.1 (R Project for Statistical Computing) to conduct statistical analyses, and MIMRGNS to calculate the average marginal effects for multiply imputed data in Stata.^43^ *P* values were 2-tailed, and statistical significance was set at less than .05.

## Results

From 3854 patients with ESRD who died in VA inpatient facilities between October 1, 2009, and September 30, 2015, we excluded 278 patients who had recovered kidney function or had a functioning kidney transplant at the time of death, 12 patients who were missing information on whether they had stopped maintenance dialysis before death or had inconsistent dates, and 195 patients whose family members were not eligible to complete the BFS. The final analytic sample included 3369 patients (mean [SD] age at death, 70.6 [10.2] years; 3320 [98.5%] male patients), of whom 1701 patients (50.5%) had a family member who responded to the BFS (eTable 1 in the Supplement). Based on information from the CMS-2746 form, 937 patients (27.8%) stopped dialysis before death, with a median (interquartile range) survival of 6 (3-11) days after their last treatment, and 2432 patients (72.2%) continued to receive dialysis treatments until they died. Documented reasons for stopping dialysis included acute complications in 334 patients (35.6%), chronic failure to thrive in 325 patients (34.7%), transplant or dialysis access failure in 10 patients (1.1%), and other reasons in 265 patients (28.3%); the reason was unknown or not reported in 3 patients (0.3%). Among a random sample of 225 patients, analysis of the CMS-2746 item on whether dialysis had been discontinued found the sensitivity was 59.2% and the specificity was 90.9%, yielding a positive predictive value of 75.0% and a negative predictive value of 82.8% (eTable 3 in the Supplement).

Compared with patients who continued dialysis, those who stopped treatment were older at death (mean [SD] age, 69.9 [10.0] years vs 72.5 [10.5] years); more likely to be white (1420 patients [58.4%] vs 604 patients [64.5%]); more likely to have received a diagnosis of dementia (362 patients [14.9%] vs 219 patients [23.4%]), cancer (667 patients [27.4%] vs 327 patients [34.9%]), or cerebrovascular disease (835 patients [34.3%] vs 363 patients [38.7%]); less likely to have received a diagnosis of myocardial infarction (804 patients [33.1%] vs 262 patients [28.0%]); and more likely to have died in a low-complexity facility (162 patients [6.7%] vs 108 patients [11.5%]) (Table). Patients who stopped dialysis were less likely than other patients to have died in facilities located in southern US census regions (Table).

**Table.** Demographic and Clinical Characteristics of Cohort

Variable	Patients, No. (%)		*P* Value
	Continued Dialysis (n = 2432)	Stopped Dialysis (n = 937)	
Age, mean (SD), y	69.9 (10.0)	72.5 (10.5)	<.001
Age group, y			
<65	799 (32.9)	242 (25.8)	<.001
65-74	852 (35.0)	292 (31.2)	
75-84	568 (23.4)	255 (27.2)	
≥85	213 (8.8)	148 (15.8)	
Male	2401 (98.7)	919 (98.1)	.21
Race			
Black	950 (39.1)	300 (32.0)	<.001
White	1420 (58.4)	604 (64.5)	
Other	62 (2.5)	33 (3.5)	
Next of kin			
Spouse or partner	1109 (45.6)	422 (45.0)	.94
Child	694 (28.5)	264 (28.2)	
Sibling	321 (13.2)	125 (13.3)	
Other	308 (12.7)	126 (13.4)	
Comorbidity			
Diabetes^a^	1829 (75.2)	688 (73.4)	.31
Congestive heart failure	1723 (70.8)	638 (68.1)	.13
Myocardial infarction	804 (33.1)	262 (28.0)	.005
Chronic obstructive pulmonary disease	1317 (54.2)	476 (50.8)	.09
Liver disease^a^	643 (26.4)	219 (23.4)	.07
Cerebrovascular disease	835 (34.3)	363 (38.7)	.02
Peripheral vascular disease^b^	1370 (56.3)	524 (55.9)	.86
Dementia^b^	362 (14.9)	219 (23.4)	<.001
Cancer^a^	667 (27.4)	327 (34.9)	<.001
Time from cohort entry to death, median (IQR), mo	50.4 (24.7-85.9)	55 (26.6-87.5)	.10
Time from ESRD onset to death, median (IQR), mo	37.1 (15.6-72.5)	39.4 (15.8-72.2)	.45
Modality			
Hemodialysis	2346 (96.5)	900 (96.1)	.66
Peritoneal dialysis	81 (3.3)	36 (3.8)	
Unknown	5 (0.2)	1 (0.1)	
Region			
New England	46 (1.9)	29 (3.1)	<.001
Mid-Atlantic	303 (12.5)	141 (15.0)	
North Central			
East	289 (11.9)	134 (14.3)	
West	166 (6.8)	112 (12.0)	
South Atlantic	670 (27.5)	168 (17.9)	
South Central			
East	188 (7.7)	67 (7.2)	
West	306 (12.6)	89 (9.5)	
Mountain	141 (5.8)	79 (8.4)	
Pacific	323 (13.3)	118 (12.6)	
Facility complexity			
High^c^	2270 (93.3)	829 (88.5)	<.001
Low^d^	162 (6.7)	108 (11.5)	
Admitted to the hospital for ≥2 wk in last 90 d before death	1423 (58.5)	534 (57.0)	.45
Admitted to ICU in last 30 d before death	1386 (57.0)	435 (46.4)	<.001
Underwent an intensive procedure in last 30 d before death	1143 (47.0)	263 (28.1)	<.001
Death setting			
ICU	1123 (46.2)	167 (17.8)	<.001
Acute care ward	703 (28.9)	179 (19.1)	
Nursing home	282 (11.6)	210 (22.4)	
Dedicated palliative care and hospice unit	324 (13.3)	381 (40.7)	
Received hospice services before death	430 (17.7)	544 (58.1)	<.001

Abbreviations: ESRD, end-stage renal disease; ICU, intensive care unit; IQR, interquartile range.

^a^ Combination of mild and severe Charlson Comorbidity Index diagnostic categories.

^b^ Charlson Comorbidity Index diagnostic category expanded as previously described.^21^

^c^ Includes level 1a, level 1b, and level 1c facilities.

^d^ Includes level 2 and level 3 facilities.

Patients who stopped dialysis were more likely than those who continued treatment to have been receiving hospice services at the time of death (544 patients [58.1%] vs 430 patients [17.7%]), less likely to have been admitted to the ICU (435 patients [46.4%] vs 1386 patients [57.0%]) or to have undergone an intensive procedure (263 patients [28.1%] vs 1143 patients [47.0%]) in the last 30 days of life, and less likely to have died in the ICU (167 patients [17.8%] vs 1123 patients [46.2%]).

### Stopping Dialysis and Family Ratings of End-of-Life Care

**Figure 2.**
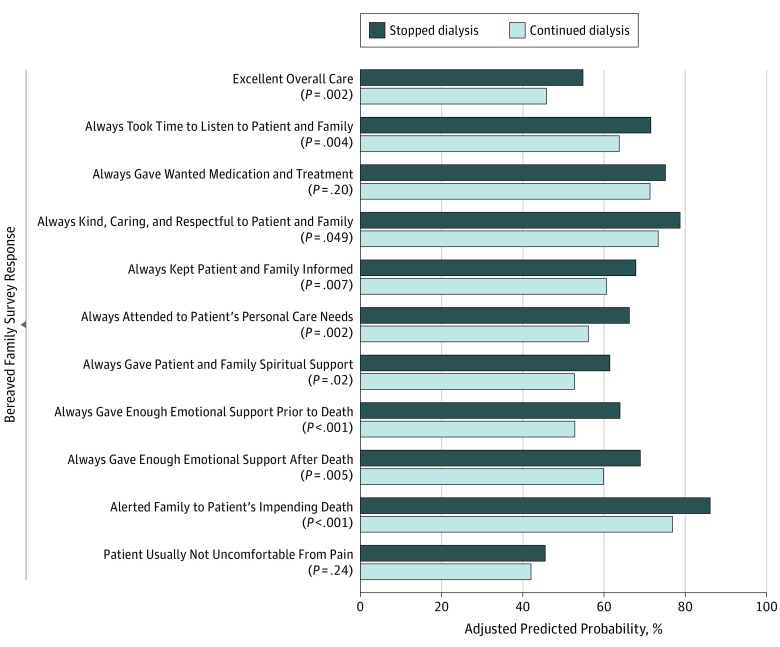
Average Marginal Effect of Stopping Dialysis on Best Responses to the Bereaved Family Survey Patients who continued dialysis were used as the reference in logistic regression, adjusted for race, age, sex, next of kin, comorbidities, region, facility complexity, year of death, high-intensity treatment, hospice services at time of death, and interaction term for hospice and stopping dialysis and weighted for unit nonresponse. Missing items were imputed, and SEs were adjusted for facility-level clustering.

Among 1701 patients with BFS responses from family members (50.5%), unadjusted family ratings of overall quality of end-of-life care and all individual items were higher for patients who stopped dialysis before death than for those who continued treatment (eTable 4 in the Supplement). In adjusted analyses, the predicted probability of patients’ family rating overall quality of care as excellent was 54.9% for patients who had stopped dialysis vs 45.9% for patients who had continued dialysis (risk difference [RD], 9.0 [95% CI, 3.3-14.8]; *P* = .002), and 8 of the 10 individual items examined were more favorable for patients who stopped dialysis (Figure 2). Specifically, family members of patients who stopped dialysis were more likely than those of patients who continued treatment to report that clinicians always took time to listen (71.6% vs 63.8%; RD, 7.8 [95% CI, 2.5-13.1]; *P* = .004); were always kind, caring, and respectful (78.8% vs 73.4%; RD, 5.5% [95% CI, 0.0%-10.9%]; *P* = .049); always kept the patient and family informed (67.9% vs 60.7%; RD, 7.2% [95% CI, 2.0%-12.4%]; *P* = .007); always attended to personal care needs (66.3% vs 56.2%; RD, 10.2% [95% CI, 3.7%-16.6%]; *P* = .002); always gave enough spiritual support (61.5% vs 52.8%; RD, 8.8% [95% CI, 1.6%-15.9%]; *P* = .02); always gave enough emotional support before death (64.0% vs 52.9%; RD, 11.2% [95% CI, 5.0%-17.4%]; *P* < .001) and after death (69.0% vs 59.9%; RD, 9.1% [95% CI, 2.8%-15.5%]; *P* = .005); and alerted them to the patient’s impending death (86.2% vs 76.9%; RD, 9.3% [95% CI, 4.8%-13.8%]; *P* < .001). There were no statistically significant differences between groups in family reports of whether clinicians always gave wanted medication and treatment (75.2% vs 71.4%; RD, 3.7% [95% CI, −2.0% to 9.5%]; *P* = .20) or in the frequency of uncontrolled pain (never or sometimes having pain: 45.6% vs 42.1%; RD, 3.5 [95% CI, −2.4 to 9.4]; *P* = .24).

**Figure 3.**
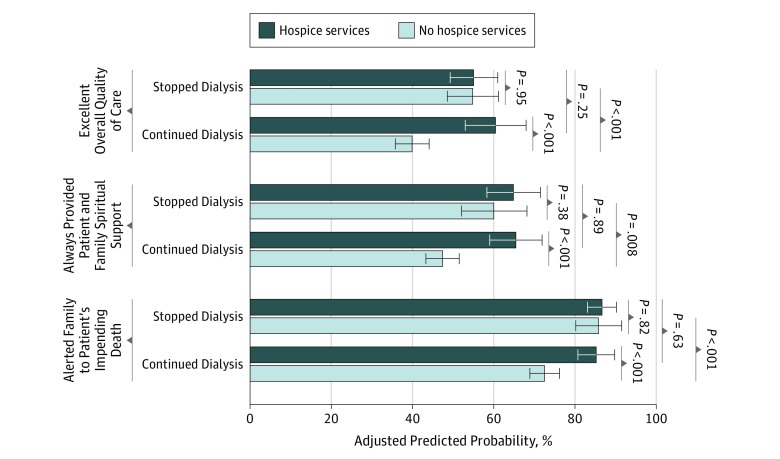
Interaction Effect of Receipt of Hospice Services and Stopping Dialysis on Best Responses to Bereaved Family Survey Items Patients who continued dialysis were used as the reference in logistic regression, adjusted for race, age, sex, next of kin, comorbidities, region, facility complexity, year of death, high-intensity treatment, hospice services at time of death, and interaction term for hospice and stopping dialysis and weighted for unit nonresponse. Missing items were imputed, and SEs were adjusted for facility-level clustering. Error bars indicate 95% CI.

There was evidence that the association between stopping dialysis and family-rated overall quality of end-of-life care (*P* for interaction = .002), spiritual support (*P* for interaction = .048), and being alerted to the patient’s impending death (*P* for interaction = .04) varied by receipt of hospice services (Figure 3; eTable 5 in the Supplement). Among patients who stopped dialysis, we found similar ratings regardless of whether patients received hospice services for overall quality of care (hospice services: 55.1% vs no hospice services: 54.9%; RD, 0.3% [95% CI, −8.3% to 9.9%]; *P* = .95), spiritual support (hospice services: 64.9% vs no hospice services: 60.1%; RD, 4.7% [95% CI, −5.8% to 15.3%]; *P* = .38), and whether clinicians alerted family to the patient’s impending death (hospice services: 86.7% vs no hospice services: 85.9%; RD, 0.8% [95% CI, −6.0% to 7.5%]; *P* = .82). However, among patients who did not stop dialysis, families of those who received hospice services were more likely than those of other patients to report that the overall rating of end-of-life care was excellent (60.5% vs 40.0%; RD, 20.5% [95% CI, 12.2%-28.9%]; *P* < .001), that clinicians always provided spiritual support (65.5% vs 47.5%; RD, 18.0% [95% CI, 10.5%-25.5%]; *P* < .001), and that clinicians alerted them to the patients’ impending death (85.3% vs 72.6%; RD, 12.7% [95% CI, 6.8%-18.6%]; *P* < .001).

## Discussion

This survey study found that among a national cohort of patients with ESRD undergoing maintenance dialysis, family-rated quality of end-of-life care was higher for patients who stopped dialysis before death than for those who continued treatment. Among patients who did not stop dialysis, ratings of overall quality of end-of-life care were substantially higher for the minority who received hospice services before death. These findings suggest that decisions to stop dialysis and to receive hospice services mark important care transitions that are associated with family-rated quality of end-of-life care for patients with ESRD.

Available evidence suggests that contemporary practices regarding stopping life-sustaining treatments, such as maintenance dialysis, may be more reflective of clinician- and system-level factors than of patient preferences.^4,44,45,46,47^ Prior studies have shown that clinicians often present intensive treatments as a default option required to prolong life and tend not to discuss the option of withdrawing life support and transitioning to comfort-oriented care.^4,46,47^ Dialysis treatment, which is often initiated in a crisis when clinical decision-making tends to be driven by medical necessity,^48^ is no exception to this rule. Prior studies in this population have suggested that many patients are unaware of their life expectancy and probable future illness trajectory,^4,49,50,51,52^ and may not even realize that stopping dialysis is an option.^4,47,49^ Furthermore, most patients undergoing dialysis do not have advance directives that address their preferences related to stopping dialysis treatments.^53,54^ Thus, decisions about stopping dialysis tend to occur late in the course of illness at times of crisis, when all other treatment options have been exhausted and there is little question that the patient is dying.^4,19,23,47,55^ In this context, stopping dialysis is likely to occur as part of a broader shift toward a more comfort-oriented approach to care. Supportive of this possibility, ratings of overall care, spiritual support, and whether families were alerted to the patient’s impending death were equally favorable for patients who stopped dialysis, regardless of whether they received hospice services. Furthermore, among those who received hospice services, ratings were equally favorable regardless of whether patients stopped dialysis. These findings suggest there is a need for more systematic efforts to prepare patients undergoing dialysis and their families for end-of-life decision-making and to inform them about the option of stopping dialysis if their illness progresses or their goals change.^50^ This might include ongoing efforts to elicit patients’ goals and values as they pertain to dialysis and other life-prolonging treatments, as well as palliative and hospice care services, and to formulate a plan for future care that reflects what is most important to each individual.^50,56,57,58,59^

Patients who die in VA facilities should theoretically not be affected by Medicare restrictions on the concurrent delivery of dialysis and hospice services. Nevertheless, stopping dialysis was associated with receipt of hospice services among members of this cohort at a rate similar to that of the overall Medicare dialysis population.^20,23,60,61^ Among patients in our study who continued dialysis until death, only 18% received hospice services, compared with 58% of patients who stopped dialysis. Strikingly, among patients who continued dialysis, there was a difference of more than 20 percentage points between those who were and were not receiving hospice services at the time of death in the proportion of family members reporting excellent overall quality of care (61% vs 40%). To our knowledge, the magnitude of this difference far exceeds between-group differences reported in previous studies using the BFS.^24,29^ For example, Kutney-Lee et al^29^ reported a 14% adjusted absolute difference in overall quality of end-of-life care for black vs white veterans, and Ersek et al^24^ reported a 12–percentage point difference in the same measure for patients with non–small cell lung cancer receiving aggressive care who died in dedicated hospice or palliative care units vs other inpatient settings. These more favorable family ratings of quality of end-of-life care for patients who continued dialysis and received hospice services suggest the need for further research on whether efforts to address barriers to concurrent receipt of dialysis and hospice services may improve quality of end-of-life care for patients with ESRD and their families. Our findings suggest there is need for more detailed descriptive research to understand how decisions to enroll in hospice and discontinue dialysis treatments occur in clinical settings and how patients on dialysis and their families could be better supported as they face these and other treatment decisions toward the end of life.

### Limitations

Our study has several limitations. First, this was an observational study, and our results cannot support causal inferences. Second, the mortality follow-back approach does not account for clinical uncertainty about whether and when patients will die.^62,63^ Nevertheless, this approach expands our understanding of what happens to patients at the end of life while addressing the significant challenges involved in engaging dying patients and their families in research.^21,64,65^ Third, while this study offers a unique perspective on the association of stopping dialysis with quality of end-of-life care, our results may not be generalizable to patients who did not die in VA facilities and to groups that were poorly represented in our cohort.^21^ Fourth, although families often provide extensive caregiving and decision-making support at the end of life,^66^ family member reports of quality of end-of-life care do not always align with what is important to the patients themselves. Fifth, we lacked detailed information about the clinical context, including the extent of patient vs family involvement in decision-making and the timing of dialysis discontinuation and hospice enrollment in relation to other treatment decisions.^23^ Sixth, consistent with earlier work, reporting of whether dialysis had been discontinued on the CMS-2746 form for members of our cohort was an insensitive marker of documentation of dialysis discontinuation in patients’ health records. However, since there is no reason to expect that errors in reporting on the CMS-2746 form would be associated with bereaved family ratings of end-of-life care, this misclassification would likely have biased our findings toward the null.^67^ Seventh, although we included data sources from VA, VA Fee Basis, and Medicare claims,^21^ our analyses did not capture episodes of care covered by Medicare Advantage or private health insurance and thus may have underestimated the intensity of end-of-life care for some cohort members. Eighth, although the BFS response rate reported in this study was comparable to that of other similar studies^68,69^ and our analyses were adjusted for nonresponse bias, our results might be subject to bias by unmeasured differences between patients whose family members did and did not respond to the BFS.

## Conclusions

In this survey study among a national cohort of patients undergoing dialysis who died in VA facilities, family members of patients who stopped maintenance dialysis before death or continued dialysis treatments but received concurrent hospice services reported more favorable ratings of end-of-life care than those of other patients. These results suggest that more effort is needed to prepare patients undergoing dialysis and their families for end-of-life decision-making, including whether and when to stop dialysis. Our findings also raise the question of whether reducing barriers to hospice enrollment for those who are receiving dialysis may help to improve the quality of end-of-life care among patients with ESRD.

## Notes

Supplement. **eAppendix.** Methods for Handling Missing Data **eTable 1.** Bereaved Family Survey Item Missingness **eTable 2.** Comparison of Respondent vs Nonrespondent Patient Characteristics **eTable 3.** Validation of the US Centers for Medicare & Medicaid Services (CMS)-2746 End-Stage Renal Disease Death Notification Form **eTable 4.** Association of Dialysis Treatment Status With Best Response on Bereaved Family Survey Items **eTable 5.** Interaction Effect of Hospice and Stopping Dialysis on Best Responses to Bereaved Family Survey Items **eReferences.**
