# Supplementary material for: Association of Family Ratings of Quality of End-of-Life Care With Stopping Dialysis Treatment and Receipt of Hospice Services
Source: JAMA Netw Open. 2019 Oct 11;2(10):e1913115. doi: 10.1001/jamanetworkopen.2019.13115 (PMC6804019; doi:10.1001/jamanetworkopen.2019.13115)
Supplement: Supplement. — eAppendix. Methods for Handling Missing Data eTable 1. Bereaved Family Survey Item Missingness eTable 2. Comparison of Respondent vs Nonrespondent Patient Characteristics eTable 3. Validation of the US Centers for Medicare & Medicaid Services (CMS)-2746 End-Stage Renal Disease Death Notification Form eTable 4. Association of Dialysis Treatment Status With Best Response on Bereaved Family Survey Items eTable 5. Interaction Effect of Hospice and Stopping Dialysis on Best Responses to Bereaved Family Survey Items eReferences. [file jamanetwopen-2-e1913115-s001.pdf]

Supplementary Online Content

Richards CA, Hebert PL, Liu C-F, et al. Association of family ratings of quality of end-of-life care with stopping dialysis treatment and receipt of hospice services. *JAMA Netw Open*. 2019;2(10):e1913115. doi:10.1001/jamanetworkopen.2019.13115

eAppendix. Methods for Handling Missing Data

eTable 1. Bereaved Family Survey Item Missingness

eTable 2. Comparison of Respondent and Nonrespondent Patient Characteristics

eTable 3. Validation of the US Centers for Medicare & Medicaid Services (CMS)-2746 End-Stage Renal Disease Death Notification Form

eTable 4. Association of Dialysis Treatment Status With Best Response on Bereaved Family Survey Items

eTable 5. Interaction Effect of Hospice and Stopping Dialysis on Best Responses to Bereaved Family Survey Items

eReferences.

This supplementary material has been provided by the authors to give readers additional information about their work.

© 2019 Richards CA et al. *JAMA Network Open.*

eAppendix. Methods for Handling Missing Data

We adjusted for survey and item non-response by non-response weighting and multiple imputation by chained equations (MICE), as previously described.[2,4–6] We believe that our assumption that data is missing at random is plausible because we have included numerous auxiliary variables listed below from several data sources (VA's Veteran Experience Center, VA clinical and administrative files, VA fee basis files [VA purchased care], Medicare inpatient and outpatient claims, and United States Renal Data System) that are associated with both missingness and overall rating of care.[7]

a. Unit non-response (Survey non-response)

Variables were included if they had an independent association with survey response at a significance of ≤ 0.2: stopped dialysis, age, race, relationship of next of kin, specific diagnoses (cancer, congestive heart failure, coronary arterial disease, diabetes, dyslipidemia, human immunodeficiency virus, liver disease, paroxysmal supraventricular tachycardia), intensive care unit (ICU) admission and intensive procedure in last 30 days, setting of death (ICU, acute ward, dedicated hospice and palliative care unit, or nursing home), mechanical ventilation and tracheostomy in the last 30 days, hospice services at death, chaplain contact prior to death, palliative care consultation in the last 90 days of life, 2 or more weeks spent in the hospital in the last 90 days, quintile of hospital days in the last 90 days, quantile of ICU days in the last 30 days, census region, facility complexity, and fiscal year of death.

b. Item non-response (Missing items for those who responded)

Variables were included if they were theoretically associated with response or overall rating of care or were in subsequent models: all above variables, in addition to all bereaved family survey items,[8] gender, quantile of Quan score,[9] dementia,[10] peripheral vascular disease, hemiplegia or paraplegia, cerebrovascular disease, chronic pulmonary disease, rheumatic disease, peptic ulcer, metastatic cancer, acute myocardial infarction, debility or failure to thrive, quintile of response probability, enteral nutrition, and feeding tube placement in last 30 days, and 2 x 2 interactions for stopping dialysis and hospice services at death, facility complexity, chaplain visit prior to death, and palliative care consult in the last 90 days. MICE was performed with 20 burn-in iterations and 40 imputations.

© 2019 Richards CA et al. *JAMA Network Open.*

eTable 1. Bereaved Family Survey Item Missingness

Bereaved Family Survey Item	Missing, No. (%)[a]
Overall rating of patient's care in the last month of life	33 (2)
Staff willing to take time to listen	47 (3)
Staff provided the treatment that patient and family wanted	65 (4)
Staff were kind, caring, respectful	28 (2)
Staff kept patient and family informed	29 (2)
Personal care needs were taken care of	98 (6)
Provided patient and family spiritual support	64 (4)
Provided patient and family emotional support prior to death	46 (3)
Provided patient and family emotional support after death	61 (3)
Staff alerted family before the patient's death	176 (10)
Patient's pain usually did not make him/her uncomfortable[b]	257 (15)

[a]Denominator includes 1,701 respondents.

© 2019 Richards CA et al. *JAMA Network Open.*

eTable 2. Comparison of Respondent vs Nonrespondent Patient Characteristics

Variables	No. (%)		*P* value
	**Respondent (n = 1701)**	**Non-Respondent (n = 1668)**	
Decisions to Stop Dialysis			<0.001
Continued Dialysis	1178 (69.3)	1254 (75.2)	
Stopped Dialysis	523 (30.7)	414 (24.8)	
Age, Mean (SD), y	71.5 (10.2)	69.7 (10.1)	<0.001
Age Group, y			<0.001
<65	479 (28.2)	562 (33.7)	
65-74	556 (32.7)	588 (35.3)	
75-84	458 (26.9)	365 (21.9)	
85+	208 (12.2)	153 (9.2)	
Male sex	1680 (98.8)	1640 (98.3)	0.35
Race			<0.001
Black	563 (33.1)	687 (41.2)	
White	1095 (64.4)	929 (55.7)	
Other	43 (2.5)	52 (3.1)	
Next of Kin			<0.001
Spouse/Partner	940 (55.3)	591 (35.4)	
Child	377 (22.2)	581 (34.8)	
Sibling	202 (11.9)	244 (14.6)	
Other	182 (10.7)	252 (15.1)	
Comorbidities			
Diabetes Mellitus[b]	1253 (73.7)	1264 (75.8)	0.17
Congestive Heart Failure	1227 (72.1)	1134 (68.0)	0.01
Myocardial Infarction	538 (31.6)	528 (31.7)	1.00
Chronic Obstructive Pulmonary Disease	895 (52.6)	898 (53.8)	0.50
Liver Disease[b]	413 (24.3)	449 (26.9)	0.09
Cerebrovascular Disease	619 (36.4)	579 (34.7)	0.33
Peripheral Vascular Disease[a]	974 (57.3)	920 (55.2)	0.23
Dementia[a]	295 (17.3)	286 (17.1)	0.92
Cancer[b]	517 (30.4)	477 (28.6)	0.27
Time from cohort Entry to Death, Median (IQR), mo	51.6 (24.8 to 86.1)	51.5 (25.5 to 86.5)	0.47
Time from onset of ESRD to Death, Median (IQR), mo	37.1 (16.1 to 71.4)	38.2 (15.2 to 73.4)	0.76
Modality			0.95
Hemodialysis	1640 (96.4)	1606 (96.3)	
Peritoneal Dialysis	58 (3.4)	59 (3.5)	
Unknown	3 (0.2)	3 (0.2)	
Region			0.51
New England	34 (2.0)	41 (2.5)	
Mid-Atlantic	209 (12.3)	235 (14.1)	
East North Central	214 (12.6)	209 (12.5)	
West North Central	156 (9.2)	122 (7.3)	
South Atlantic	429 (25.2)	409 (24.5)	
East South Central	133 (7.8)	122 (7.3)	
West South Central	199 (11.7)	196 (11.8)	
Mountain	107 (6.3)	113 (6.8)	

© 2019 Richards CA et al. *JAMA Network Open.*

Pacific	220 (12.9)	221 (13.2)	
Facility Complexity			0.07
High[c]	1550 (91.1)	1549 (92.9)	
Low[d]	151 (8.9)	119 (7.1)	
Admitted to the hospital for ≥2 wk in last 90 d	957 (56.3)	1000 (60.0)	0.03
Admitted to ICU in last 30 d before death	882 (51.9)	939 (56.3)	0.01
Underwent an intensive procedure in last 30 d before death	666 (39.2)	740 (44.4)	0.002
Death Setting			<0.001
ICU	603 (35.4)	687 (41.2)	
Acute care ward	433 (25.5)	449 (26.9)	
Nursing Home	278 (16.3)	214 (12.8)	
Dedicated Palliative Care and Hospice Unit	387 (22.8)	318 (19.1)	
Received hospice services before death	541 (31.8)	433 (26.0)	<0.001

Abbreviations: ESRD, end-stage renal disease; ICU, intensive care unit.
[a]Charlson Comorbidity Index diagnostic category expanded.
[b]Combination of Charlson mild and severe diagnostic categories
[c]Includes level 1a, level 1b, and level 1c facilities.
[d]Includes level 2 and level 3 facilities.

© 2019 Richards CA et al. *JAMA Network Open.*

eTable 3. Validation of the US Centers for Medicare & Medicaid Services (CMS)-2746 End-Stage Renal Disease Death Notification Form

		Electronic Health Record (Gold Standard)		
		**Stopped Dialysis**	**Continued Dialysis**	**Total**
**CMS-2746 Form**	**Stopped Dialysis**	42	14	56
	**Continued Dialysis**	29	140	169
	**Total**	71	154	225

© 2019 Richards CA et al. *JAMA Network Open.*

eTable 4. Association of Dialysis Treatment Status With Best Response on Bereaved Family Survey Items

**Bereaved Family Survey Item**[a]	**Undjusted Model** *Denominator varies due to missing items*[b]				**Adjusted Model**[c] (*N* = 1701)[d]			
	**Stopped Dialysis**[e]		**Risk Difference (95% CI)**[f]	***P* value**[f]	**Stopped Dialysis**[e]		**Risk Difference (95% CI)**[f]	***P* value**[f]
	**No (%)**	**Yes (%)**			**No (%)**	**Yes (%)**		
Excellent overall quality of care	44.5	56.8	12.4 (7.0 to 17.7)	<0.001	45.9	54.9	9.0 (3.3 to 14.8)	0.002
Staff always took time to listen	63.0	74.2	11.2 (6.4 to 16.0)	<0.001	63.8	71.6	7.8 (2.5 to 13.1)	0.004
Staff always gave wanted medication & treatment	70.8	77.6	6.8 (2.6 to 11.1)	0.002	71.4	75.2	3.7 (-2.0 to 9.5)	0.20
Staff were always kind, caring, respectful	73.4	81.7	8.3 (4.2 to 12.5)	<0.001	73.4	78.8	5.5 (0.0 to 10.9)	0.049
Staff always kept patient & family informed	59.7	70.8	11.1 (7.0 to 15.2)	<0.001	60.7	67.9	7.2 (2.0 to 12.4)	0.007
Staff always attended to personal care needs	54.8	66.6	11.8 (6.8 to 16.9)	<0.001	56.2	66.3	10.2 (3.7 to 16.6)	0.002
Staff always gave enough spiritual support	51.7	62.6	10.9 (5.1 to 16.8)	<0.001	52.8	61.5	8.8 (1.6 to 15.9)	0.02
Staff always gave enough emotional support before death	51.8	66.8	15.0 (10.5 to 19.5)	<0.001	52.9	64.0	11.2 (5.0 to 17.4)	<0.001
Staff always gave enough emotional support after death	59.2	70.2	11.0 (5.9 to 16.0)	<0.001	59.9	69.0	9.1 (2.8 to 15.5)	0.005
Staff alerted family before the patients' death	75.6	84.4	8.8 (4.7 to 13.0)	<0.001	76.9	86.2	9.3 (4.8 to 13.8)	<0.001
Patient's pain usually didn't make him/her uncomfortable[g]	43.0	49.3	6.3 (1.2 to 11.5)	0.02	42.1	45.6	3.5 (-2.4 to 9.4)	0.24

[a]Dichotomized best response vs. all other responses.
[b]Denominator varies, eTable 1 for proportion of missing items.
[c]Model adjusted for race, age, gender, next of kin, region, facility complexity, year of death, comorbidities, 2+ wks spent in hospital in last 90 d of life, ICU admission in last 30 d, intensive procedure in last 30 d, death in ICU, hospice services at the time of death, and interaction term for hospice services and stopping dialysis, weighted for non-response; [d]missing items imputed in adjusted models.
[e]Presented are the predicted probabilities over the distribution of covariates in the respondent sample.
[f]95% confidence intervals and *P* values are for the differences in predicted probabilities, standard errors adjusted for clustering in facilities.
[g]Pain was dichotomized as "never" or "sometimes" vs. "always" or "usually."

© 2019 Richards CA et al. *JAMA Network Open.*

eTable 5. Interaction Effect of Hospice and Stopping Dialysis on Best Responses to Bereaved Family Survey Items

**Bereaved Family Survey Item**[a]	**Adjusted Model**[b] **(N = 1,701)**[c]			
	**Stopped Dialysis**		**Risk Difference (95% CI)**[e]	***P* value**[e]
	**No (%)**[d]	**Yes (%)**[d]		
Excellent overall quality of care				
Received hospice services before death	60.5	55.1	-5.3 (-14.5 to 3.8)	0.25
No hospice services	40.0	54.9	14.9 (7.2 to 22.6)	<0.001
Staff always gave enough spiritual support				
Received hospice services before death	65.5	64.9	-0.6 (-9.1 to 7.9)	0.89
No hospice services	47.5	60.1	12.6 (3.3 to 22.0)	0.008
Staff alerted family to patient's impending death				
Received hospice services before death	85.3	86.7	1.4 (-4.2 to 7.0)	0.63
No hospice services	72.6	85.9	13.3 (7.0 to 19.6)	<0.001

[a]Dichotomized best response vs. all other responses.
[b]Adjusted for race, age, gender, next of kin, region, facility complexity, year of death, comorbidities, 2+ wks spent in hospital in last 90 d of life, ICU admission in last 30 d, intensive procedure in last 30 d, death in ICU, hospice services at the time of death, interaction term for hospice services and stopping dialysis, weighted for non-response.
[c]Missing items imputed.
[d]presented are the predicted probabilities over the distribution of covariates in the respondent sample.
[e]95% confidence intervals and *P* values are for the differences in predicted probabilities, standard errors adjusted for clustering in facilities.

© 2019 Richards CA et al. *JAMA Network Open.*

eReferences.

1. von Elm E, Altman DG, Egger M, Pocock SJ, Gotzsche PC, Vandenbroucke JP. The Strengthening the Reporting of Observational Studies in Epidemiology (STROBE) Statement: guidelines for reporting observational studies. http://www.equator-network.org/reporting-guidelines/strobe/.

2. Richards CA, Liu C, Hebert PL, et al. Family perceptions of quality of end-of-life care for veterans with advanced chronic kidney disease. *Clin J Am Soc Nephrol*. In Press;14(9).

3. American Association for Public Opinion Research. Survey disclosure checklist. https://www.aapor.org/Standards-Ethics/AAPOR-Code-of-Ethics/Survey-Disclosure-Checklist.aspx. Published May 13, 2009.

4. White IR, Royston P, Wood AM. Multiple imputation using chained equations: issues and guidance for practice. *Stat Med*. 2011;30(4):377-399. doi:10.1002/sim.4067

5. Chen Q, Gelman A, Tracy M, Norris FH, Galea S. Incorporating the sampling design in weighting adjustments for panel attrition. *Stat Med*. 2015;34(28):3637-3647. doi:10.1002/sim.6618

6. Little R. Survey nonresponse adjustments for estimates of means. *Int Stat Rev*. 1986;54(2):139-157. doi:10.2307/1403140

7. Sterne JAC, White IR, Carlin JB, et al. Multiple imputation for missing data in epidemiological and clinical research: potential and pitfalls. *BMJ*. 2009;338(jun29 1):b2393-b2393. doi:10.1136/bmj.b2393

8. Thorpe JM, Smith D, Kuzla N, Scott L, Ersek M. Does mode of survey administration matter? Using measurement invariance to validate the mail and telephone versions of the bereaved family survey. *J Pain Symptom Manage*. 2016;51(3):546-556. doi:10.1016/j.jpainsymman.2015.11.006

9. Quan H, Sundararajan V, Halfon P, et al. Coding algorithms for defining comorbidities in ICD-9-CM and ICD-10 administrative data. *Med Care*. 2005;(43):1130–1139.

10. Fujiyoshi A, Jacobs DR, Alonso A, Luchsinger JA, Rapp SR, Duprez DA. Validity of death certificate and hospital discharge ICD codes for dementia diagnosis: the multi-ethnic study of atherosclerosis. *Alzheimer Dis Assoc Disord*. 2017;31(2):168-172. doi:10.1097/WAD.0000000000000164

© 2019 Richards CA et al. *JAMA Network Open.*
